# Race-Related Differences Between and Within Sex to Experimental Thermal Pain in Middle and Older Adulthood: An Exploratory Pilot Analysis

**DOI:** 10.3389/fpain.2021.780338

**Published:** 2021-11-17

**Authors:** Karen O. Moss, Kathy D. Wright, Alai Tan, Karen M. Rose, Douglas W. Scharre, Tanya R. Gure, Ronald L. Cowan, Michelle D. Failla, Todd B. Monroe

**Affiliations:** ^1^College of Nursing, The Ohio State University, Columbus, OH, United States; ^2^Center for Healthy Aging, Self-Management and Complex Care, College of Nursing, The Ohio State University, Columbus, OH, United States; ^3^Center for Health Outcomes in Medicine, Scholarship and Service (HOMES), College of Medicine, The Ohio State University, Columbus, OH, United States; ^4^Discovery Themes-Chronic Brain Injury Program, The Ohio State University, Columbus, OH, United States; ^5^Center for Cognitive and Memory Disorders, The Ohio State University Wexner Medical Center, Columbus, OH, United States; ^6^College of Medicine, The Ohio State University, Columbus, OH, United States; ^7^Division of General Internal Medicine and Geriatrics, The Ohio State University Wexner Medical Center, Columbus, OH, United States; ^8^Department of Psychiatry, College of Medicine, University of Tennessee Health Sciences Center, Memphis, TN, United States

**Keywords:** experimental pain, psychophysics, race, sex, thermal pain

## Abstract

This brief report details a pilot analysis conducted to explore racial differences in pain sensitivity and unpleasantness between cognitively healthy Black and White adults, stratified by sex. A total of 24 cognitively healthy adults (12 Black and 12 White) from two completed studies were matched by age and sex, and divided into two groups based on race. Stratified analyses by sex demonstrated that Black females reported experiencing pain intensity ratings of all three intensity sensations at lower temperatures than White females. These findings will inform future research studies to determine if these results hold true in a fully-powered sample and should include mixed methodologies, incorporating neuroimaging data to further assess this phenomenon. Improving pain assessment and management across racial/ethnic groups will help healthcare providers such as nurses and physicians to ensure optimal quality of life for all.

Pain is a complex and poorly managed international public health problem (1). According to the International Association for the Study of Pain's revised definition, pain is “an unpleasant sensory and emotional experience associated with, or resembling that associated with, actual or potential tissue damage” (2). Therefore, the pain experience is measured in multidimensional modes, including sensory (intensity) and affective (unpleasantness) components (3).

Sex-differences in pain perceptions for men and women exist in both clinical and experimental pain studies with women generally reporting greater pain sensitivity and unpleasantness relative to men (4, 5). When examining differences by race, Blacks report more pain compared to Whites, irrespective of age or pain type [reviewed in (6, 7)]. A seminal study conducted by Portenoy et al. found that non-Hispanic Blacks and Hispanics reported more severe pain than Whites (8). Additionally, among nursing home residents living with cancer who consumed equivalent analgesics, behavioral pain reports were significantly higher among Blacks relative to Whites (9). Most literature on sex, race, and pain in adults is based upon self-report or observational techniques (10) that examine these variables separately (11) with no studies, to our knowledge, focused on the combined effects of race and sex on experimental pain.

Older Blacks, in particular, have more pain producing chronic conditions, such as arthritis, and other geriatric syndromes that are likely associated with severe pain (e.g., depressive symptoms, immobility) than Whites (12). Racial and ethnic differences also exist in pain management (7). Therefore, Blacks are at high risk for greater pain experiences that are underreported and undermanaged (7). Limited psychophysical findings thus far indicate that Blacks also experience lower pain tolerance (increased sensitivity) and higher pain ratings (more intensity) than Whites [reviewed in (6)]. Specifically, there is a need to better understand differences in central pain mechanisms and psychophysical response to pain among Blacks in order to improve their pain management and subsequently their quality-of-life (6). This information would be especially applicable for older Blacks who are at greatest risk for undermanaged pain (13). The aim of this pilot study was to explore racial differences in pain sensitivity and unpleasantness between cognitively healthy Black and White adults, stratified by sex.

## Materials and Methods

### Study Participants

Participants were selected from two completed experimental pain studies (NIH/NIA K23 AG046379 and NIH/NIA R21 AG049332) using very similar psychophysical procedures and behavioral data. First, the total number of Blacks available for inclusion was 13 yet one did not have complete data leaving a total of (*n* = 12) Black adults with complete data. We then purposively selected 12 White participants matched on age and sex (see demographics in Table 1). Thus, the final sample consisted of 24 cognitively healthy adults matched by age and sex, and divided into two groups based on race.

**Table 1:** Sample characteristics and racial difference in pain by sex.

	**Female**						**Male**					
	**White** **(*****n*** **=** **6)**		**Black** **(*****n*** **=** **6)**		**White vs. Black**		**White** **(*****n*** **=** **6)**		**Black** **(*****n*** **=** **6)**		**White vs. Black**	
	**Mean**	**SD**	**Mean**	**SD**	**Cohen's D**	**P**	**Mean**	**SD**	**Mean**	**SD**	**Cohen's D**	**P**
**Sample characteristics**												
Age	65.3	12.2	65.2	10.7	0.02	0.980	63.0	8.8	62.7	11.2	0.04	0.955
Body Mass Index	27.2	5.3	24.0	6.7	0.57^++^	0.390	28.4	4.9	33.1	6.4	−0.91^++^	0.182
Socioeconomic Status	48.5	9.7	43.9	18.1	0.35^+^	0.597	52.9	10.9	51.7	5.8	0.15	0.865
Trait Anxiety	47.5	3.0	49.7	4.1	−0.66^++^	0.320	46.5	2.5	46.8	3.7	−0.12	0.859
**Intensity pain ratings (Medoc**© **Q-sense)**												
Just Noticeable	33.2	1.5	31.8	0.4	1.35^+++^	0.078	34.7	1.4	34.7	1.2	0.00	1.000
Weak/Mild	40.3	2.3	35.0	2.2	2.58^+++^	0.002	38.5	3.5	39.8	3.3	−0.43^+^	0.510
Moderate	44.8	2.8	41.5	2.7	1.32^+++^	0.063	43.0	3.2	43.3	4.1	−0.10	0.878
**Unpleasantness pain scores (0–20 Scale)**												
Just Noticeable	0.3	0.5	1.5	3.2	−0.56^++^	0.418	0.8	1.0	1.7	1.5	−0.72^++^	0.283
Weak/Mild	3.3	2.3	2.5	2.6	0.38^+^	0.565	5.5	0.8	4.2	2.8	0.71^++^	0.305
Moderate	8.3	2.3	7.3	3.8	0.35^+^	0.597	9.5	1.4	8.0	5.0	0.45^+^	0.505

*Effect size*:

+ *Small (0.2 ≤ Cohen's d < 0.5)*;

++ *Medium (0.5 ≤ d < 0.8)*;

+++ *Large (d ≥ 0.8)*.

### Measurements

#### Experimental Pain Psychophysics

Experimental pain data were collected using the Medoc© Q-Sense (14) thermal stimulus delivery system to assess the perceptions of “just noticeable,” “weak/mild,” and “moderate” pain intensity applied to the right thenar eminence. The baseline temperature was 30 degrees Celsius (°C) with a ramp rate of 4°C/s. After each perception, unpleasantness scores were measured using a 0–20 scale, with 0 indicating no pain unpleasantness and 20 indicating worst pain unpleasantness (15). A series of three trials of each perception were completed and the average temperature (intensity) ratings and unpleasantness scores recorded.

#### Anxiety

Trait anxiety was measured with the 20-item Spielberger Trait Anxiety Inventory (16) which focuses on anxiety proneness (20 items) with scores ranging from 20 to 80 for each subscale with higher scores indicating greater anxiety (16, 17).

#### Socioeconomic Status (SES)

The Hollingshead four-factor index of SES was used to measure SES (18). The Hollingshead SES scale measures marital, retirement, education, and occupation status (18).

#### Body Mass Index (BMI)

Because BMI can influence the pain experience (19), BMI was calculated on each participant to ensure there were no between group differences that might confound the results.

### Protocol

This cross-sectional study occurred over 2 days in the parent studies. The protocol details can be found in Monroe et al. (20). The first day consisted of a screening and enrollment/consenting visit and the second day consisted of all other data collection procedures listed above, including psychophysics. Each visit lasted ~1 h. Participants were reimbursed $100.00/day for their time. All procedures were approved by the Vanderbilt University Institutional Review Board.

### Statistical Analysis

Descriptive statistics were used to summarize sample characteristics and pain ratings, by sex (female vs. male) and race (White vs. Black). In each sex stratum, differences between the racial groups were compared by: (1) means and standard deviations (SDs), (2) two-sample *t*-tests, and (3) effect sizes (Cohen's *d*). A *p* < 0.05 was considered statistically significant. The Cohen's *d*-values of 0.2, 0.5, and 0.8 were cutoffs for small, medium, and large effect sizes, respectively. All analyses were conducted using SAS 9.4 (SAS® Institute, Cary, North Carolina).

## Results

Table 1 includes the sample characteristics by race and sex. Mean age for Black participants was 63.9 years (SD: 10.5) and 64.2 years (SD: 10.2) for White participants. There were no significant between-group differences in age. White females had higher BMI (*d* = 0.57), and lower trait anxiety (*d* = −0.66) than Black females, while White males had lower BMI (*d* = −0.91) than Black males. Stratified analyses by sex demonstrated that Black females reported experiencing pain intensity ratings of all three intensity sensations at lower temperatures than White females, especially for “weak/mild pain” (mean ± SD: 35.0 ± 2.2 vs. 40.3 ± 2.3, *p* = 0.002 (21), *d* = 2.58), respectively. In contrast, Black males reported similar intensity pain ratings at all three intensity sensations as White males. There were no statistically significant differences for unpleasantness scores. However, Black participants reported higher scores for “just noticeable” pain unpleasantness and lower scores for “weak/mild” and “moderate” pain unpleasantness. These differences were of small to medium effect sizes with absolute temperature differences at 0.9–1.5 degrees (Table 1).

## Discussion

Though a relatively small sample, the current exploratory pilot results support and extend previously conducted research on race, sex, and pain. Established literature indicates that females are generally more sensitive to pain, have more pain related diagnoses, and report pain as more intense (5). However, the focus of most research on sex differences in pain does not include race as a primary predictor variable and similarly most research on racial differences in pain does not include within sex as a group comparison (5, 7).

Current pilot results support the notion that the interaction of race and sex may have important effects when designing future studies and preparing clinical practice guidelines. Overall, the current study demonstrated patterns of experimental pain responses, similar to previous research (6, 7). Similar to overall findings by race from Portenoy et al., when subdivided by sex in the current study, Black females were more sensitive to pain than White females (21). Although preliminary, the relationship between pain differences among Black and White females was robust. However, our sample size did not have sufficient power to detect statistically significant associations for effect size that are smaller than 1.8. For example, the racial differences of just noticeable unpleasantness pain scores were of medium to large effect sizes for both females and males (d = −0.56 and −0.72, respectively), even though the differences did not reach statistical significance (*p* = 0.42 and 0.28, respectively). Therefore, we reported both effect sizes and statistical *p*-values to guide results interpretation. Because of the small size we did not adjust for demographic and behavioral covariates (e.g., Age, SES, BMI, anxiety).

These pilot findings will inform future research that should seek to determine whether these findings are supported in a larger study including multiple psychophysical tests (e.g., threshold, tolerance, temporal summation, conditioned pain modulation) and multiple experimental pain measures (e.g., thermal, mechanical pressure, cold pressure, electrical shock). In addition to added experimental psychophysical procedures, more studies are needed to determine the impact and presence of geriatric co-morbidities that affect pain perception and intensity, which may differ by race and sex (e.g., cancer, diabetes, osteoarthritis). Thus, additional experimental and clinical studies of pain are needed in larger samples to provide more evidence to inform assessment and treatment of pain in racially diverse groups of male and female adults.

Because this study included mostly older adults and were purposefully age-matched, generalizability of findings is limited. To improve our understanding of the pain experience and pain mechanisms in this population, future studies using neuroimaging and mixed-methods approaches are needed.

Assessment and management of pain can be optimized when healthcare providers, such as nurses and physicians are able to better understand pain intensity and unpleasantness differences in adults from diverse sex and racial backgrounds. This is a necessary first step to ensure optimal quality of life through improvements in pain management for all adults, regardless of their sex or race.

## Data Availability Statement

The datasets used and/or analyzed during the current study are available from the senior author on reasonable request. Requests to access these datasets should be directed to Todd Monroe (monroe.1181@osu.edu).

## Ethics Statement

The studies involving human participants were reviewed and approved by Vanderbilt University Institutional Review Board. The patients/participants provided their written informed consent to participate in this study.

## Author Contributions

KM: investigation, writing—original draft, writing—review and editing, visualization, and project administration. KW, KR, DS, TG, and MF: writing—review and editing and visualization. AT: formal analysis, resources, data curation, writing—review and editing, and visualization. RC: conceptualization, methodology, validation, writing—review and editing, visualization, and funding acquisition. TM: conceptualization, methodology, validation, investigation, resources, writing—original draft, visualization, supervision, project administration, and funding acquisition. All authors contributed to the article and approved the submitted version.

## Funding

Data accessed for this project were combined from studies supported by the National Institutes of Health, National Institute on Aging projects K23 AG046379 and R21 AG049332.

## Conflict of Interest

The authors declare that the research was conducted in the absence of any commercial or financial relationships that could be construed as a potential conflict of interest.

## Publisher's Note

All claims expressed in this article are solely those of the authors and do not necessarily represent those of their affiliated organizations, or those of the publisher, the editors and the reviewers. Any product that may be evaluated in this article, or claim that may be made by its manufacturer, is not guaranteed or endorsed by the publisher.
